# A novel thermophilic chitinase directly mined from the marine metagenome using the deep learning tool Preoptem

**DOI:** 10.1186/s40643-022-00543-1

**Published:** 2022-05-16

**Authors:** Yan Zhang, Feifei Guan, Guoshun Xu, Xiaoqing Liu, Yuhong Zhang, Jilu Sun, Bin Yao, Huoqing Huang, Ningfeng Wu, Jian Tian

**Affiliations:** 1 Biotechnology Research Institute, Chinese Academy of Agricultural Sciences, Beijing, 100081 China; 2 College of Food Science and Technology, Hebei Agricultural University, Baoding, 071000 Hebei China; 3 Institute of Animal Science, Chinese Academy of Agricultural Sciences, Beijing, 100193 China

**Keywords:** Deep learning, Chitinase, Thermal stability, Chitooligosaccharides

## Abstract

Chitin is abundant in nature and its degradation products are highly valuable for numerous applications. Thermophilic chitinases are increasingly appreciated for their capacity to biodegrade chitin at high temperatures and prolonged enzyme stability. Here, using deep learning approaches, we developed a prediction tool, Preoptem, to screen thermophilic proteins. A novel thermophilic chitinase, Chi304, was mined directly from the marine metagenome. Chi304 showed maximum activity at 85 ℃, its *T*_m_ reached 89.65 ± 0.22℃, and exhibited excellent thermal stability at 80 and 90 °C. Chi304 had both endo- and exo-chitinase activities, and the (GlcNAc)_2_ was the main hydrolysis product of chitin-related substrates. The product yields of colloidal chitin degradation reached 97% within 80 min, and 20% over 4 days of reaction with crude chitin powder. This study thus provides a method to mine the novel thermophilic chitinase for efficient chitin biodegradation.

**Graphical Abstract:**

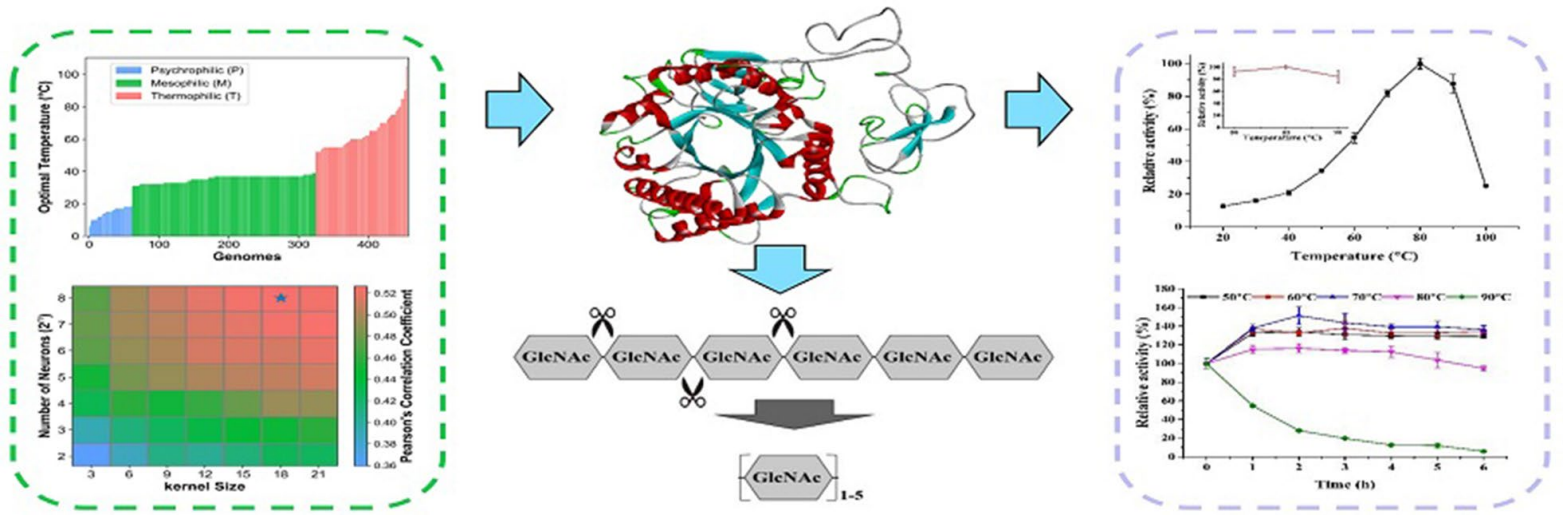

**Supplementary Information:**

The online version contains supplementary material available at 10.1186/s40643-022-00543-1.

## Introduction

Chitin, a polysaccharide polymer linked by N-acetyl-D-glucosamine (GlcNAc) through β-1,4-glycosidic bonds, is the second most abundant component of biomass in nature (Dash et al. 2011). It is estimated that the natural biosynthesis of chitin is nearly 100 billion tons annually (Yang et al. 2016). Chitinases, which are glycoside hydrolases, play an essential role in the hydrolysis of glycosidic bonds in chitin to form soluble chitooligosaccharides (Barad et al. 2020). These products have been adopted for agricultural uses in regulating plant immunity and defense systems, enhancing plant response to biotic stress, promoting plant growth and yield and improving fruit quality and shelf-life (Desaki et al. 2018; Jia et al. 2020; Pusztahelyi 2018).

Chitin has a highly ordered crystalline structure and is insoluble in water. Commonly used degradation or pretreatment methods include chemical, physical and enzymatic methods. Chemical method generally refers to the treatment of chitin with strong acid, which will cause serious environmental pollution (Yabushita et al. 2015). Physical methods include high temperature, microwave and ultrasonic wave, etc. (Ajavakom et al. 2012). Although this method avoids environmental pollution, the degradation efficiency of chitin is low. While the enzyme method has the advantages of green, efficiency and environmental friendliness, the dense structure of chitin makes it not fully accessible to enzymes, which can be alleviated if chitin has undergone some degree of pretreatment, such as high temperature. So high-temperature chitinases and their thermal stability are important for use in the conversion of waste chitin powder to the high value-added production of chitooligosaccharides. While most chitinases exhibited their highest activity at an optimum temperature below 65 ℃ (Loni et al. 2014; Yahiaoui et al. 2019), only a few thermophilic chitinases such as the ChiA-Mt45 (Mohamed et al. 2019), ChiA-Hh59 (Bouacem et al. 2018), ChiA (Tanaka et al. 1999), and GlcNase (GlmATk) (Tanaka et al. 2003) have been reported. Their optimum temperatures were 90, 85, 85, and 80 ℃, respectively. However, these four acidic chitinases only acted on colloidal chitin or chitooligosaccharides, and their activities on crude chitin powder were unknown and remained only at the level of scientific research. Kuzu et al. detected the activity of chitinase in the culture supernatant of *Bacillus thuringiensis *subsp. *kurstaki* HBK-51, isolated from chitin-containing wastes, with an optimum reaction temperature of 110 ℃. To the best of our knowledge, this represents the highest recorded temperature for chitin degradation. Unfortunately, the nucleic acid sequence of the responsible chitinase and the purified enzyme were not published (Kuzu et al. 2012).

Most studies such as the four chitinases mentioned above rely on finding candidate thermophilic chitinases from culturable thermophilic microorganisms. However, this method is greatly limited since most microorganisms cannot yet be isolated from the natural environment by culture-based techniques, especially those in extreme environments. Moreover, chitinase may be rare among culturable thermophilic microorganisms, which increases the difficulty of finding novel thermophilic chitinases. However, extreme environmental samples (oceans, lakes, hydrothermal springs, forests, etc.) not only contain huge microbial resources, but also a wealth of bioactive molecules and enzymes. Metagenome- and genome-mining methods (function-based or sequence based, etc.) that do not rely on culturable microorganisms can thus exploit these resources at a larger scale and explore deeper levels of metabolic capability in environmental samples (Adam and Perner 2018; Alma'abadi et al. 2015; Mirete et al. 2016; Nasseri et al. 2018). Additionally, the number of sequenced metagenomes in public databases has grown substantially in recent years, and which have become more accessible to researchers with the development of functional annotation methods, such as Pfam (El-Gebali et al. 2019), CAZY (Palcic 2011) and BLAST (Huerta-Cepas et al. 2017). However, until now a few method has been developed to reliably predict detailed temperature-related properties of proteins, which can be used in conjunction with functional annotation tools to directly screen thermophilic proteins in metagenomic data.

One promising approach for predictive annotation of protein properties is deep learning, a sub-field of machine learning that uses multi-layered deep neural networks (DNNs) to extract novel features from classified input sequences. Deep learning is generally most effective when applied to large datasets. The essence of most machine learning algorithms is to find patterns in the available data and heavily rely on data (Siedhoff et al. 2020; Singh et al. 2021). In the enzyme engineering field, there are only a few published models predicting the optimal temperature of enzymes. In 2019, Engqvist et al. reported a machine learning model to accurately predict the optimal growth temperature for bacteria, archaea and microbial eukaryotes and further predicted the catalytic temperature optima of enzymes with the machine learning model. Thus the catalytic temperature optima of 6.5 million enzymes, covering 4447 enzyme classes were predicted successfully (Li et al. 2019). In addition, another machine learning model, TOME (temperature optima for microorganisms and enzymes), was developed by Payne in 2020. Through ensemble learning and resampling strategies, the accuracy of prediction was significantly improved (Gado et al. 2020). However, most of the current machine learning models only provided prediction methods and lacked strong experimental data.

In light of these previous studies, we hypothesize that, due to its high polymerization and density, crude chitin will be more easily biodegraded under high temperatures by novel thermophilic chitinases that maintain prolonged enzyme stability. In this study, to test this hypothesis, we developed a deep learning predictive tool, Preoptem, to predict the optimal temperate of proteins, and used it to explore a large metagenomic dataset. We subsequently screened the novel thermophilic chitinase Chi304 directly from a previously published ocean metagenome (Sunagawa et al. 2015). To validate the predictive capacity of Preoptem and to characterize the activity of Chi304, we investigated its stability at high temperatures and activity against a range of substrates, including high density and high polymerization degree crude chitin. We also screened the antimicrobial activity of the Chi304 hydrolysis products of chitin.

## Materials and methods

### General

We purchased the crude chitin powder [(C_8_H_13_NO_5_)_n_, MW: 203.19, product code: L1825062] from Aladdin (Shanghai, China), N-acetyl-D-glucosamine (GlcNAc) from YuanYe Bio-Technology (Shanghai, China), and (GlcNAc)_2–6_ from Huich Biotech (Shanghai, China) companies. The polymerases and restriction endonucleases used in this study were previously described in Guan’ work (Guan et al. 2020). Mutant sequences were synthesized by General Biosystems Ltd (Anhui, China). The LB medium (L^−1^) consisted of 10.0 g of NaCl, 5.0 g of yeast extract and 10.0 g of peptone. The pH of the LB medium was adjusted to 7.0 using NaOH. The TSA medium (L^−1^) was composed of 10.0 g of peptone, 10.0 g of saccharose and 1.0 g of glutamic acid. The pH of the TSA medium was adjusted to 7.0 with NaOH. Both solid media were amended with 1.5% agar.

The colloidal chitin was prepared according to a previously described method (Garcia-Fraga et al. 2015) with slight modifications. A total of 5 g of chitin were dissolved in 90 mL of 37% HCl and stirred vigorously at room temperature for 2 h. Subsequently, we added 410 mL of 95% pre-cooled ethanol and stirred for another 30 min, which was followed by swelling overnight at 4 ℃. The mixture was then centrifuged at 10,000 × *g* for 10 min at room temperature, and the precipitate was collected and washed with distilled water until the pH approached a neutral value. The neutral precipitate was then dissolved with distilled water to a final concentration of 2% and then stored at 4 ℃.

### Dataset

The optimal temperatures for each of the microorganisms were collected from the BacDive database (Söhngen et al. 2014). As most of the microorganisms in the database belong to the mesophilic class, we selected those with optimal temperatures < 20 °C, > 30 °C and < 40 °C, and > 50 °C, as the psychrophilic, mesophilic and thermophilic organisms, respectively. In the case of the mesophilic class, microorganisms belonging to different genera were selected in order to reduce the number of genome number and increase the gene complexity of this class. However, in the case of the psychrophilic, and thermophilic classes, the microorganisms with different species were selected in the dataset, as these two classes had fewer number of species than that of the mesophilic class. Importantly, we only selected the species that were fully sequenced and annotated in the database. As a result, a total of 456 species were collected and their genomes downloaded from the NCBI (ftp://ftp.ncbi.nlm.nih.gov/genomes/Bacteria/) database, as shown in Additional file 1: Table S1.

The program Ortholog Finder 2 (ver. 2.2.7) (Emms and Kelly 2019) was used to identify the orthologs among all of the proteins in the three classes (psychrophilic, mesophilic or thermophilic proteins). Only the proteins in the homolog cluster with at least two classes were selected for training the prediction models, as the method was able to make model predictions about the thermal property of the proteins.

### Model construction

We employed deep leaning models trained on the TensorFlow platform and based on Keras 2.0.1 in a Python 2.7 programming environment. We leveraged previously reported deep learning methods from other fields and used open-source code, with some modifications. We first transformed the protein sequence as the one-Hot-Coded matrix. The final architecture comprised two convolutional layers, each followed by a maximum pooling layer; two fully connected layers, each followed by a dropout layer (dropout rate of 0.3); and a final prediction layer. In this model, we used a “relu” activation function (except for the final prediction layer). In order to optimize the parameters, we used the optimizer adaptive moment estimation (Adam) with the AMSGRAD parameter as true. The MSE (mean squared error) served as both loss function and performance metric. The maximum training epoch and batch size of the training were set as 100 and 32, respectively. We employed the early stopping technique in the training process in order to avoid over-training and subsequent over-fitting of the prediction model. The tenfold cross-validation was applied to split the homologous clusters, train and validate the performance of the proposed method.

### Protein expression and purification

The chitinase Chi304 encoding sequence was synthesized by General Biosystems Co., Ltd (Anhui, China) and linked to the pET30a vector at *Eco*RI (5′) and *Xho*I (3′). The recombinant plasmid was then transferred into the *E. coli* expression strain BL21(DE3), with a random single clone being picked and cultured as a seed solution. 2 mL of the seed solution was then transferred to 200 mL of LB medium containing kanamycin (at a final concentration of 50 μg/mL) and cultured at OD_600_ = 0.6–0.8 at 37 ℃, 200 rpm. Subsequently, we added isopropyl-β-D-thiogalactopyranoside (IPTG) at a final concentration of 0.25 mM and reduced the culture temperature to 16 ℃ in order to induce protein expression for 16–18 h. The culture mixture was then centrifuged at 4 ℃, 13, 786 × *g* for 5 min, and the bacterial precipitation was harvested and resuspended in a Tris–HCl buffer (20 mM, pH 8.0). The mixture was then lysed through ultrasonication for 5 min (every 5-s interval, ultrasound 3 s, 10 times in total) on ice. Subsequently, we obtained the supernatant by centrifugation at 4 ℃ and 13,786 × *g* for 30 min. From the crude enzyme solution, we purified Chi304 using a Ni column, as illustrated in a previous work (Guan et al. 2020).

### Detection of degraded activity to colloidal chitin

The activity of Chi304 was determined by measuring the amount of reducing sugars released during chitin hydrolysis with colloidal chitin as the substrate. The reaction mixture (1 mL) consisted of 250 μL Chi304 (25 μg/mL), 250 μL 2% (w/v) colloidal chitin and 500 μL glycine–NaOH buffer (100 mM, pH 9.0). After incubation at 80 °C for 30 min, 1 mL of 3,5-dinitrosalicylic acid (DNS) was added in order to terminate the reaction (Additional file 2: Fig. S1). This was followed by boiling for 10 min and cooling to room temperature. The mixture was then centrifuged for 1 min at 13, 786 × *g* and the supernatant used to detect the absorbance at 540 nm (Miller 1959). A total of three experimental replicates were conducted. One unit of chitinase activity was defined as the amount of chitinase used to yield 1 μmol of reducing sugars per minute under the aforementioned conditions.

### Enzymatic properties

In order to determine the optimal pH values necessary to ensure maximum Chi304 activity and stability, we used various reaction buffers (Na_2_HPO_4_–citric acid buffer, pH 3.0–8.0; Tris–HCl buffer, pH 8.0–9.0; and glycine–NaOH buffer, pH 9.0–11.0). The reaction system used for determining the optimum pH was 250 μL Chi304 (25 μg/mL), 250 μL colloidal chitin and 500 μL reaction buffer with different pH values. The pH stability assay was performed by pre-incubating Chi304 (0.25 mg/mL) on ice for 1 h in different buffer solution at different pH values (pH 3.0–11.0). The residual enzyme activity was then detected as described above.

After detecting the optimum Chi304 temperature, we selected a temperature in the range of 20–90 ℃. The specific procedure consisted of 250 μL Chi304 (25 μg/mL) mixed with 250 μL colloidal chitin and 500 μL glycine–NaOH buffer (100 mM, pH 9.0), after which the system was incubated at different temperatures for 30 min. Chi304 temperature stability was determined by measuring its residual activity after incubating at 50, 60, 70, 80 and 90 ℃ for 1, 2, 3, 4, 5 and 6 h, respectively. The residual enzyme activity was then calculated according to the method described above.

### Chitin pretreatment and detection by scanning electron microscopy

A total of 10 mL ddH_2_O was added to a tube containing 20 mg of crude chitin powder. The mixture was consecutively treated by ultrasonic homogenizer (JY92-IIN, 25 kHz, 260 W, China, 20 min), numerically controlled ultrasonic cleaner (KQ-300DE, 40 ℃, 40 kHz, 120 W, China, 20 min), microwave (2 min) and water bath at 80 ℃ (8 h). Crude chitin powder without any treatment was used as control. Colloidal chitin and crude chitin powder that was enzymolyzed by Chi304 were also prepared. All samples were freeze-dried. Scanning electron microscopy (SEM, Hitachi SU8010, Tokyo, Japan) was performed in order to investigate the surface of the pretreated chitin (Zhang et al. 2018).

### Detection of degraded activity to chitin oligosaccharides and crude chitin powder

In cases where the degradation substrates were (GlcNAc)_2_, (GlcNAc)_3_ or (GlcNAc)_6_, the reactions were performed by incubating 10 μL Chi304 (2 mg/ml), 20 μL glycine–NaOH buffer (100 mM, pH 9.0) and 10 μL chitooligosaccharides (50 mM). The reaction system was then incubated at 80℃ for 10 min, 30 min, 1 h, 2 h, 5 h, 8 h, and 12 h.

In contrast, in cases where the substrate was (GlcNAc)_4_ or (GlcNAc)_5_, the reaction system consisted of 10 μL Chi304 (0.1 mg/mL), 20 μL glycine–NaOH buffer (100 mM, pH 9.0) and 10 μL chitooligosaccharides (50 mM). After this, the reaction system was transferred to a water bath at 80℃ for 2 min, 5 min, 10 min, 30 min, 1 h, 3 h, and 8 h.

In cases where the degraded substrate was crude chitin powder, the reaction system was expanded to 50 mL, specifically consisting of 250 mg of crude chitin powder substrate, 25 mL Chi304 (1 mg/mL) and 25 mL glycine–NaOH buffer (100 mM, pH 9.0). The system was then placed on a magnetic stirrer with continuous stirring at 200 rpm at 80℃ for 6 h, 12 h, 24 h, 36 h, 48 h, 60 h, 72 h and 96 h.

### Detection of degradation products by thin-layer chromatography

We used a thin-layer chromatography analysis (TLC) in order to analyze the degradation products. The detailed procedure consisted of 1 μL of reaction mixture stained on silica gel plates (0.2–0.25 mm) (HPTLC Silica gel 60, Merck Co., Germany). The developing solvent [n-butanol:methanol:aqueous ammonia:water = 5:4:2:1 (v/v/v/v)] was then used to unfold the sample for about 1 h. After natural air drying, we uniformly sprayed a chromogenic agent (400 uL aniline, 400 mg diphenylamine, 20 mL acetone, and 3 mL 85% phosphoric acid) on the surface of the plate and dried for 15 min at 120 °C, after which the product was visualized (Hong et al. 2008).

### Detection of degradation products by MALDI-TOF

The tested samples consisted of 1 μL of degradation products and 1 μL of matrix. The matrix contained 20 mg α-cyano-4-hydroxycinnamic acid [HCCA] and 20 mg 2,5-dihydroxybenzoic acid [DHBA] dissolved in 2 mL of 90% methanol/water with 0.1% formic acid. The sample mixture was then subjected to matrix-assisted laser desorption ionization-time of flight mass spectrometry (MALDI-TOF MS) (Bruker Daltonics, Billerica, MA). The results were analyzed using flexAnalysis 3.4 software (Guan et al. 2020).

### Detection of degradation products by HPLC

The reaction product was filtered by a 0.22-μm filter membrane and analyzed using high-performance liquid chromatography (Shimadzu LC-20AT). The reaction conditions were as follows: chromatogram column: Shodex Sugar KS-802; column temperature: 60℃; mobile phase: H_2_O; detector: Shimadzu RIDE-20A; flow rate: 0.6 mL/min.

### Detection of the antibacterial activity of chitin oligosaccharides

The completely degraded colloidal chitin was collected by centrifuging at 13, 786 × *g* at 4 ℃ for 1 min. The supernatant was then taken for vacuum freeze-drying. The lyophilized powder was dissolved in double distilled water to different concentrations (50, 75, 100, 125 and 150 mg/mL) for later use. The Gram-positive bacteria *Bacillus subtilis* WB600 and the Gram-negative bacteria *Xanthomonas* sp. saved in our laboratory were used to examine the antibacterial activity using the disk inhibition zone assay.

*Xanthomonas* sp. was cultured overnight in TSA medium, in order to be activated. The mixtures were then separately diluted to OD_600_ = 0.3. 4% (V/V) and added to the corresponding solid medium (heat preservation at 60 ℃). After the culture medium containing the bacteria were poured onto the plate and solidified, Oxford cups were gently placed on the surface of the medium. A total of 100 μL of chitin oligosaccharide solutions at different concentrations were added to the Oxford cups on the TSA plates.

After *B. subtilis* WB600 was cultured and activated in a LB medium overnight, the bacterial solution was diluted to OD_600_ = 0.3. 0.1% (V/V) and incubated to the solid LB medium (heat preservation at 60 ℃). After preparing the plate as described above, we added 200 μL of chitin oligosaccharide solutions at different concentrations to the Oxford cup.

The plates were then gently moved to the incubator at 28 ℃ for culture. After 24 h of incubation, the diameters of the inhibition zones were measured using a Vernier caliper. All tests were performed a total of three times for each sample in order to ensure proper replication.

### Online predictor

In order to make the Preoptem predictive tool easily accessible to scientists in the protein research community, we built a new website (http://www.elabcaas.cn/pird/preoptem.html) using the Hypertext Markup Language (HTML). All models were constructed using Perl as the backend code. Users can easily predict the optimal protein temperatures using our Preoptem website.

## Results and discussion

### Construction of a deep learning model to discriminate thermophilic proteins

**Fig. 1:**
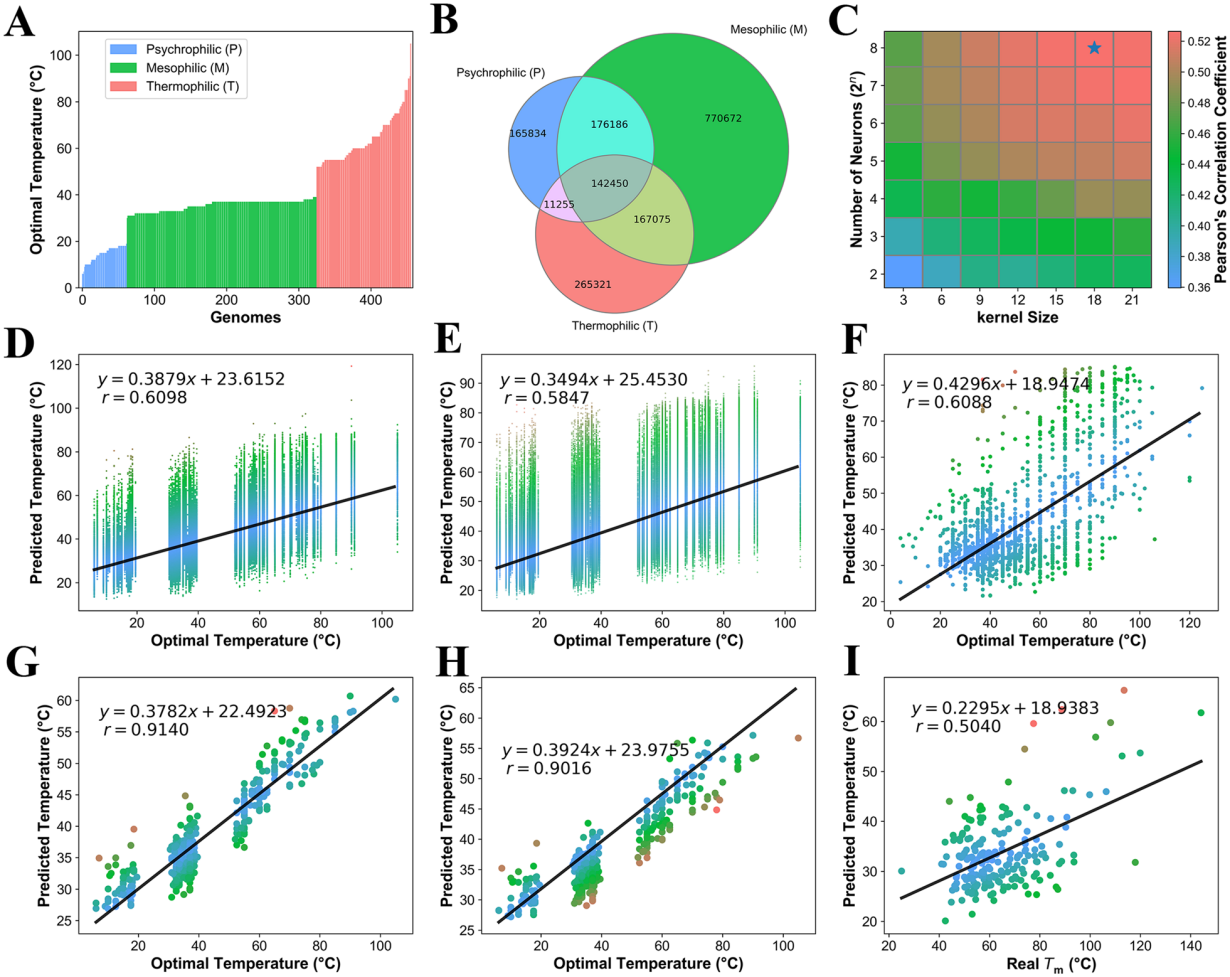
Construction of a deep learning model to screen for potential thermophilic proteins. **A** Dataset collected in this study. The x-axis represents species sorted by optimal temperature and y-axis indicates optimal temperature. **B** Venn diagrams of the psychrophilic (blue), mesophilic (green), and thermophilic (pink) proteins. **C** Optimization of the parameters of kernel size and number of neurons in the deep learning model. Star indicates the highest value of correlation coefficient. **D** Correlation between the predicted and observed optimal temperatures in the validation dataset. **E** Correlation between the predicted and observed optimal temperatures in the test dataset. **F** Correlation analysis of predicted optimal temperatures for protein with known optimal temperature in the Uniprot database. **G** Correlation between the predicted and observed optimal temperatures in the cross-validation system. **H** Correlation between the predicted and observed optimal temperatures for a protein that is not included in the training dataset. **I** Correlation analysis of predicted optimal temperatures for protein activity and experimentally determined *T*_m_. **D**–**I** The blue dot indicates that the data are close to the fitted line, the red dot indicates that the data are far away from the fitted line, and the green dot indicates that the data are centered from the fitted line

To construct the deep learning model for screening the thermophilic proteins, a protein dataset with different temperature tolerance was constructed. Firstly, the psychrophilic, mesophilic, and thermophilic microorganisms were distinguished according to their optimal growth temperature by screening the BacDive database. Then, their corresponding protein sequences of those microorganisms were downloaded from NCBI. A total of 456 different species were included in the dataset, which contained 241,416 psychrophilic, 880,211 mesophilic, and 326,547 thermophilic proteins (Additional file 1: Table S1 and Fig. 1A). As it was an unbalanced dataset and the proteins from diverse species differed in function, the proteins were then reorganized based on orthologous analysis. As shown in Fig. 1B, only 550,523 proteins among the 108,658 ortholog clusters were found in more than one of the psychrophilic, mesophilic or thermophilic classes. And those 550,523 proteins were used to train and validate the models based on the tenfold cross-validation. The other 897,651 proteins that were not used for training the models were selected as the test dataset. All of the 550,523 proteins were then encoded within the one hot encoded matrix and were given a label indicating the optimal temperature for the corresponding microorganisms from which they were derived. A tenfold cross-validation strategy was used to train and evaluate the deep learning models, in which all orthologous clusters were split into ten folds. The kernel size and number of neurons were optimized by the global search algorithm, and for which the Pearson’s correlation coefficient (*r*) between the experimentally determined and predicted optimal temperature in all of the validation datasets was used as the indicator. As a result, the highest *r* value among all models in the validation dataset reached as high as 0.61, and the optimized kernel size and neuron number were 18 and 256, respectively (Fig. 1C). The Pearson’s correlation coefficient (*r*) on the training, validation and test datasets were 0.63 ± 0.01, 0.61 ± 0.01 (Fig. 1D) and 0.58 ± 0.01(Fig. 1E), respectively. In addition to Pearson’s correlation coefficient (*r*), we also used the MAE (mean absolute error) as the other metric to evaluate the performance of the models. And the MAE on the training, validation and test datasets were 9.21 ± 0.14, 9.34 ± 0.11 and 9.62 ± 0.12 ℃, respectively. Additionally, we have downloaded all of the proteins with known optimal temperature in the Uniprot database. We predicted its optimal temperature and fit the data with the experimentally determined optimal temperature. The Pearson’s correlation coefficient (*r*) (Fig. 1F) and MAE were 0.61 ± 0.01 and 13.86 ± 0.36. The results also indicated that the method could be used to mine the thermophilic enzymes.

To further evaluate the performance of the constructed models, all of the predicted optimal temperatures of proteins from the same genome in the cross-validation system were averaged together to determine the predicted optimal temperature for that genome in the training dataset, optTem(pre1). As shown in Fig. 1G, the optTem(pre1) and the experimental optimal temperature of the microorganism, optTem(exp1), were strongly correlated, indicated by a Pearson’s correlation coefficient was 0.91. Additionally, we predicted the optimal temperatures of proteins that did not exist in the training dataset using the constructed predictive models. The predicted optimal temperatures of those proteins within the same genomes were averaged as the vector optTem (pre2). A strong correlation (Fig. 1H) was also observed between the experimental optimal temperature of the microorganism, optTem(exp2) and optTem(pre2). These results indicated that the model was robust and could be used to reliably predict optimal temperature of proteins. In addition, we also calculated the predicted optimal temperature of proteins, optTem(pro) with known *T*_m_ value. A strong correlation coefficient (*r* = 0.50) between the optTem(pro) amd *T*_m_ of proteins was also observed (Fig. 1I), indicating that this method could be used to identify thermophilic proteins. As established by earlier studies, protein structure and amino acid sequence always influences its enzymatic properties. In our database, the frequency of amino acid E, L, V, Y is significantly positively correlated with thermophilicity, while a high frequency of amino acid D, H, M, Q, S, T is negatively correlated with thermophilicity (Additional file 2: Fig. S2), in agreement with the rule reported by Igor N. Berezovsky in 2005 (Berezovsky and Shakhnovich 2005).

### Screening the thermophilic chitinase from the metagenome

**Fig. 2:**
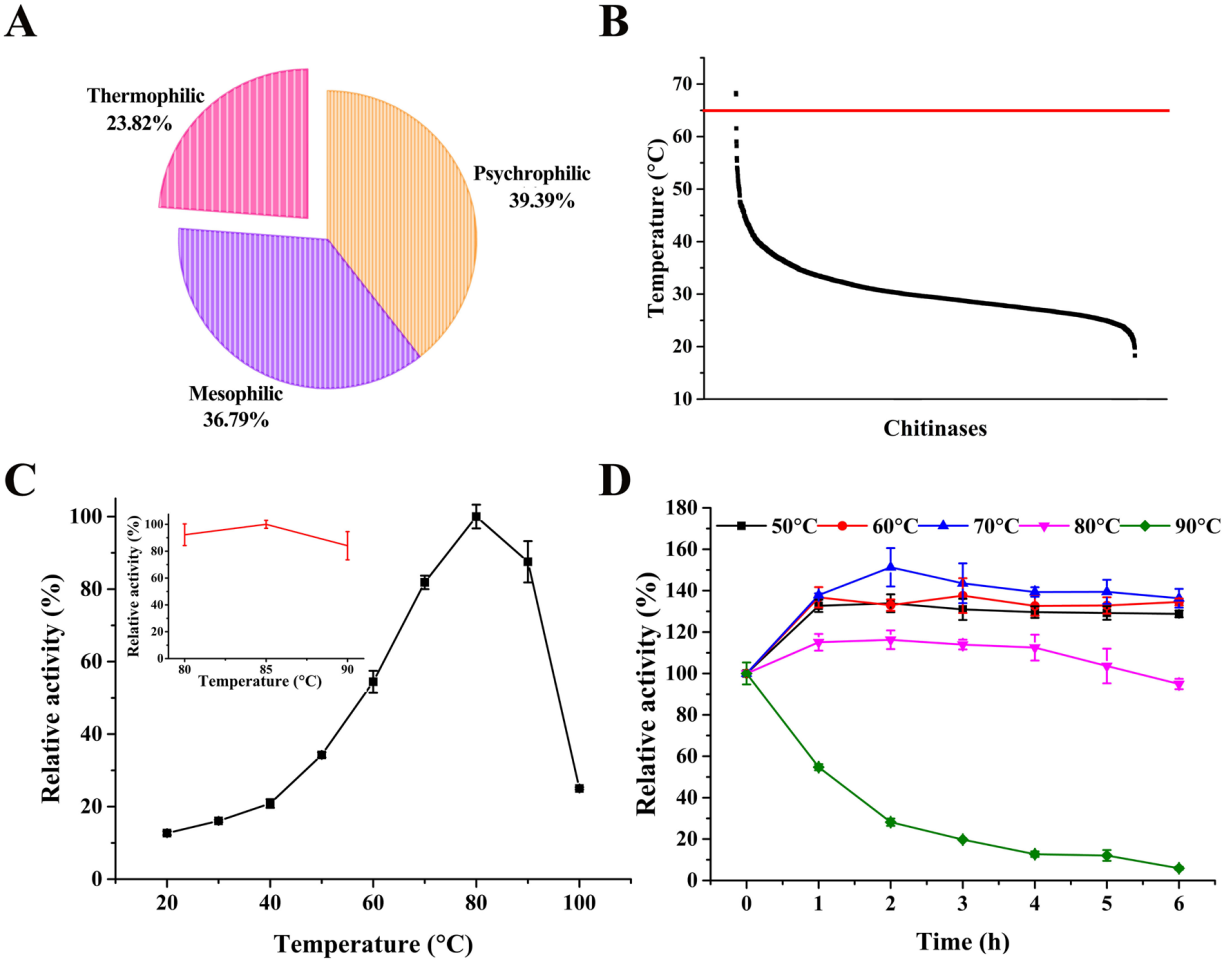
Biochemical characteristics of Chi304. **A** The number of predicted psychrophilic, mesophilic, and thermophilic proteins among the total chitinases screened from the ocean metagenome. **B** Predicted optimal temperatures of screened chitinases. **C** Optimum temperature and **D** thermal stability of selected chitinase Chi304

Next, we developed a method to screen the thermophilic chitinase from the ocean metagenome by the constructed deep learning models. The annotated protein sequences of the ocean metagenome (Sunagawa et al. 2015) were downloaded and the tool hmmsearch in the package HMMER was used to search the chitinase with the Pfam file (ID: PF00704). As a result, the 3199 proteins (Additional file 1: Table S2) were screened out from the metagenome and predicted with our deep learning models described above. As shown in Fig. 2A, approximately 39.39%, 36.79%, and 23.82% of the proteins were potentially psychrophilic, mesophilic, or thermophilic, respectively. We sorted these proteins based on their predicted optimal temperature (Fig. 2B), and the protein with the highest optTem(pre) among the full set was Chi304. The *chi304* gene (GenBank Accession Number: MW446948) was then synthesized, inserted into the expression plasmid pET30a( +), and the encoded protein was expressed by *E. coli* BL21(DE3), and subsequently purified using a Ni–NTA agarose column (Qiagen, USA). The results of SDS-PAGE gel separation showed a single band for the Chi304 protein at 70.95 kDa, which was consistent with its theoretical size (Additional file 2: Fig. S3). Its maximum specific enzyme activity towards colloidal chitin was 12.28 U mL^−1^, with a surprisingly high *T*_m_ value of 89.65 ± 0.22 ℃.

We then used colloidal chitin as substrate to determine the optimal temperature for Chi304 function and we found that starting at 20 ℃, Chi304 activity continued to increase with rising temperature until it peaked, remarkably, at 85 °C before decreasing; at 90 ℃, the protein still maintained 87% of its maximum activity (Fig. 2C). In view of the fact that there was little difference in Chi304 activity between 80 and 85 °C, we conducted all subsequent experiments at 80 °C for consistency. The Chi304 protein also exhibited very high thermal stability, with its enzymatic activity improving to varying degrees under incubation at 50–70 °C for 1–6 h, and reaching as high as 137% of its original performance after 2 h at 70 °C. Its activity also increased slightly when incubated at 80℃ for 4 h, and in the following 6 h, it maintained 95% residual enzymatic activity. Even after 1 and 2 h at 90℃, the residual enzymatic activity still reached 54% and 28% of its original activity, respectively (Fig. 2D). The optimum pH for the Chi304 reaction peaked at 9.0 under optimum temperature, and it remained active in pH range of 3.0 to 11.0 (Additional file 2: Fig. S4A). In addition, the relative residual activity of Chi304 was close to 100% that of the original after incubation on ice at pH 8.0–10.0 for 1 h (Additional file 2: Fig. S4B).

Compared to other chitinases, Chi304 showed the highest optimum temperature and thermal stability for activity under alkaline conditions yet reported for a chitinase. In addition to chitinase, we have used the tool to mine the psychrophilic catalase, thermophilic protease, thermophilic glucose oxidase and thermophilic laccase. We experimentally identified at least five functional proteins for each enzyme. Of those functional proteins, approximately the 2–3 enzymes in the five functional proteins of each enzyme are the expected enzymes. Therefore, the general success rate is around 40–60%.

### Chi304 exhibited both endo- and exo-activity

**Fig. 3:**
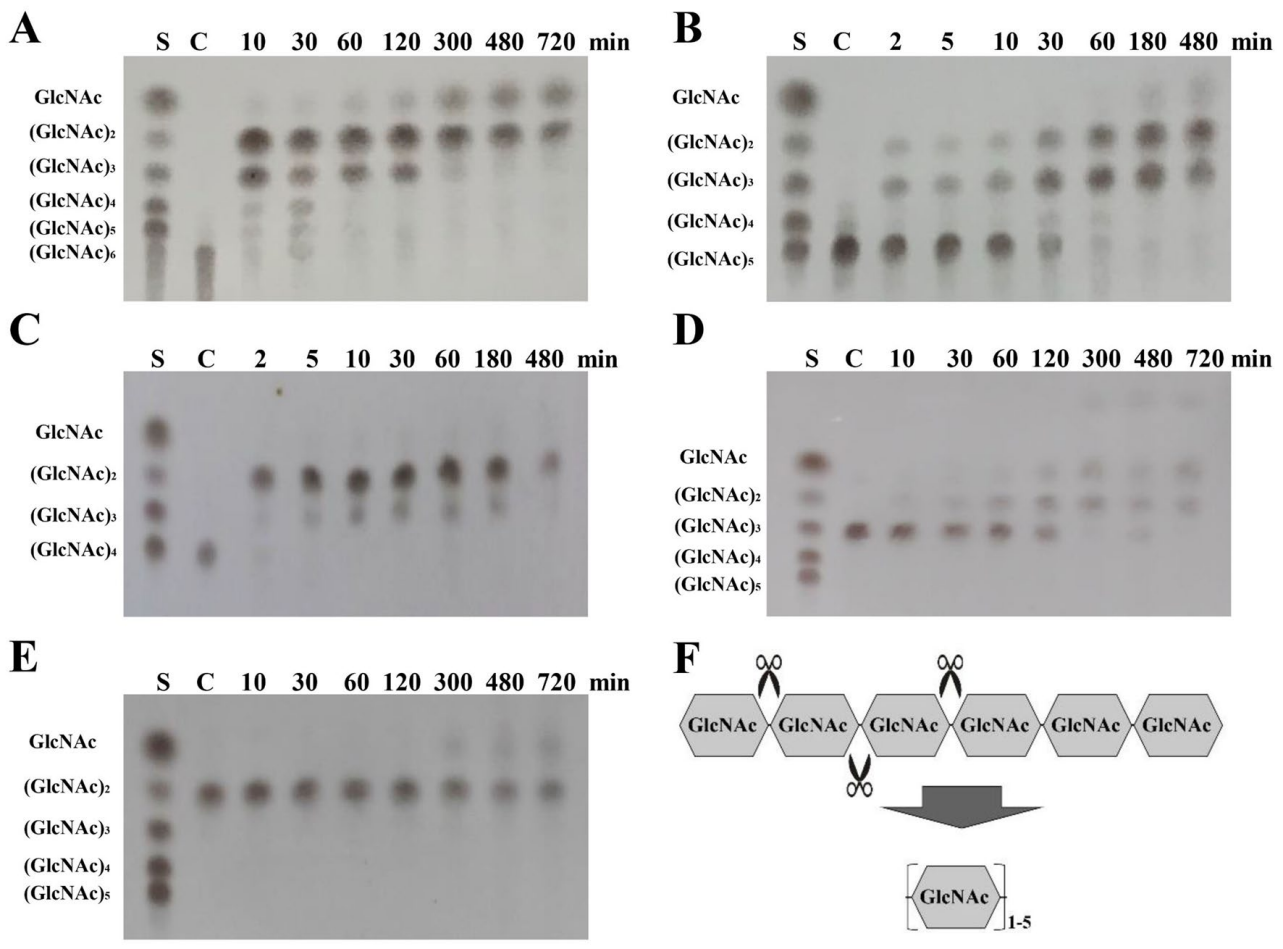
Detection of chitooligosaccharide hydrolysis products by TLC. **A** Detection of (GlcNAc)_6_ hydrolysis products. **B** Detection of (GlcNAc)_5_ hydrolysis products. **C** Detection of (GlcNAc)_4_ hydrolysis products. **D** Detection of (GlcNAc)_3_ hydrolysis products. (E) Detection of (GlcNAc)_2_ hydrolysis products. **F** Schematic diagram of chito-oligosaccharides degradation. S, 50 mg mL^−1^ (GlcNAc)_1–6_ mixed standard; C, negative control for chitinase activity

The endo-chitinases attack the chitin on random points, and the products are mainly (GlcNAc)_n_ (n ≥ 3). While the exo-enzymes act on the reducing or non-reducing end of the polysaccharide, producing GlcNAc or (GlcNAc)_2_ (Kidibule et al. 2020; Wang et al. 2016). In order to clarify the degrading mechanism of Chi304, we first measured its activity to hydrolyze chitin oligosaccharides with 2–6 degrees of polymerization. Thin layer chromatography showed that Chi304 (2 mg/mL) could almost completely degrade 50 mM (GlcNAc)_6_ into (GlcNAc)_1–5_ within 30 min (Fig. 3A), and the possible cleavage sites are shown in Fig. 3F, suggesting that Chi304 had both endo- and exo-activity; when (GlcNAc)_5_ and (GlcNAc)_4_ were used as substrates for Chi304 (0.1 mg/mL) degradation, the main products were (GlcNAc)_2_ and (GlcNAc)_3_ within 2 min (Fig. 3B and C), suggesting that the chitin oligosaccharides with polymerization degrees of 4 and 5 were the optimal substrates for Chi304 and Chi304 exhibited strong exo-activity; However, Chi304 (2 mg/mL) also had a weak ability to degrade chitin oligosaccharides with low degree of polymerization (*n* = 2 or 3). Specifically, within 720 min, (GlcNAc)_3_ was completely degraded to (GlcNAc)_2_ and GlcNAc (Fig. 3D). Within the same time range, Chi304 could only degrade a proportion of (GlcNAc)_2_ to produce GlcNAc (Fig. 3E). To sum up, Chi304 showed higher degradation activity toward chitin oligosaccharides with higher degree of polymerization (Suzuki et al. 2002) (Fig. 3F).

### Chi304 degrades colloidal chitin with high efficiency

**Fig. 4:**
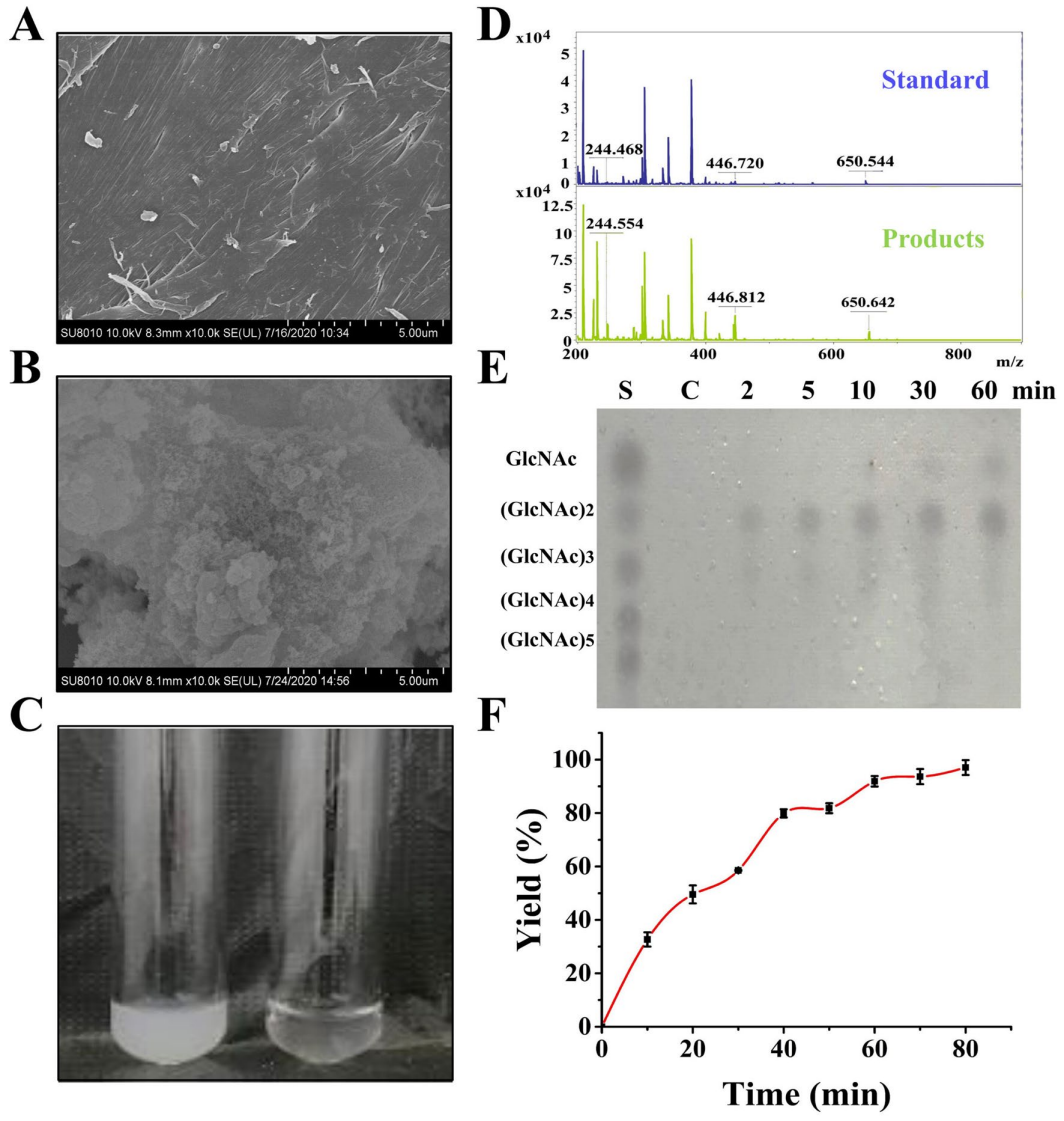
Detection of colloidal chitin degradation. **A** The surface morphology of crude chitin powder observed by SEM. **B** The surface morphology of colloidal chitin observed by SEM. **C** The changes of colloidal chitin before and after Chi304 enzymolysis. **D** MALDI-TOF detection of colloidal chitin degradation; standard, 5 mg mL^−1^ (GlcNAc)_1–6_ mixed standard. **E** Detection of colloidal chitin hydrolysis by TLC; S, 50 mg mL^−1^ (GlcNAc)_1–5_ mixed standard. **F** Yields of reducing sugar products of colloidal chitin degradation

In order to degrade crude chitin powder more efficiently, we investigated changes in the physicochemical state of the original crude chitin powder under non-enzymatic pre-treatments. The SEM results showed that, compared to the raw crude chitin power (Fig. 4A), the colloidal chitin had more damage to its highly polymerized components, and the chitin was distributed in a loose, flocculated form (Fig. 4B). The ultrasonication (20 min), ultrasonic cleaning (20 min) and microwave irradiation (2 min) resulted in less damage on the physical surface morphology of chitin (Additional file 2: Fig. S5).

In order to maximize the efficiency of chitin degradation, we tested colloidal chitin as the substrate for Chi304, since this pretreatment provided the strongest initial degradative effects on raw chitin polymer. The results showed that after 2-h exposure to Chi304, the colloidal chitin was apparently degraded, indicated by a change in its physical appearance from a cloudy suspension to clear solution (Fig. 4C). Detection by MALDI-TOF revealed that the main degradation product was (GlcNAc)_2_, accompanied by low levels of (GlcNAc)_3_ and GlcNAc (Fig. 4D). Analysis by TLC further showed that (GlcNAc)_2_ and (GlcNAc)_3_ were produced within 2 min of starting the reaction, while GlcNAc was produced after 30 min (Fig. 4E). The yields of these products over the time are shown in Fig. 4. Degradation of colloidal chitin into reducing sugars was rapid within the first hour, yielding approximately 90%, and slowed gradually over the 80 min reaction time, ultimately resulting in a 97% yield.

### Chi304 could directly degraded crude chitin powder and the chitin oligosaccharide product has definite antibacterial effects

**Fig. 5:**
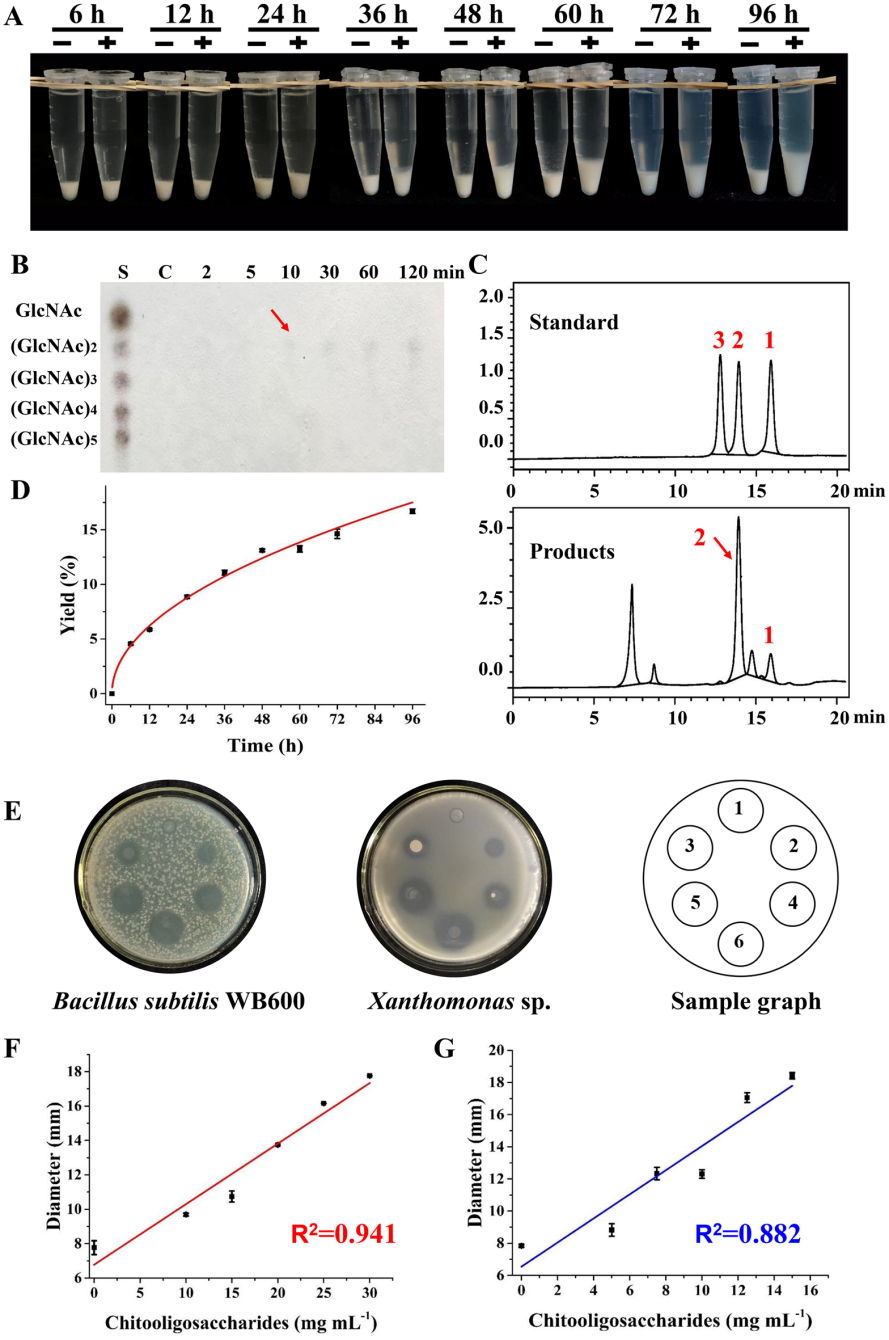
Degradation of crude chitin powder by Chi304 and the antibacterial activity of the chitooligosaccharides products. **A** Accumulation of degraded crude chitin powder under exposure to Chi304. **B** TLC detection of crude chitin powder hydrolysis; S, 50 mg mL^−1^ (GlcNAc)_1–5_ mixed standard. **C** HPLC detection of crude chitin powder hydrolysis; standard, 1 mg mL^−1^ (GlcNAc)_1–3_ mixed standard. **D** Yields of reducing sugars produced by crude chitin powder degradation. **E** Zones of inhibition produced by different concentrations of chitosan oligosaccharides. Zones 1–6 had 0, 25, 50, 75, 100, and 125 mg mL^−1^ chitosan oligosaccharides, respectively. **F**, **G** Linear relationship between chitosan oligosaccharide concentration and the diameter of inhibition zone for **F** *B. subtilis* WB600 and **G** *Xanthomonas* sp

We initially tested the capacity for Chi304 to directly degrade the comparatively recalcitrant crude chitin powder. To this end, we monitored changes in the morphology and composition of crude chitin powder exposed to Chi304 in solution at 12 h intervals over a 96-h time course. The results showed that, over time, chitin accumulated in large quantities at the bottom of the colloidal solution in the presence of Chi304, and by 96 h, the volume was approximately threefold that of the control group (Fig. 5A), possibly due to degradative changes in the chitin structure incurred by Chi304 activity. We used scanning electron microscopy imaging to observe changes in the physicochemical state of the original crude chitin powder that resulted from enzymatic hydrolysis by Chi304. The results showed that Chi304 activity caused depolymerization of the chitin, compared with the markedly lower effects of high temperature and reaction buffer (Additional file 2: Fig. S6). TLC analysis of the degradation products showed that a small amount of (GlcNAc)_2_ was produced within 10 min, and increased over the following 2 h (Fig. 5B). More sensitive detection by HPLC detection at 2 h into the reaction confirmed that the main product was (GlcNAc)_2_, as well as a small amount of GlcNAc (Fig. 5C). The degradation products were sampled at 12-h time points over the following 4 days for detection of reducing sugars, which ultimately showed that Chi304 was able to degrade 20% of the initial crude chitin over 96 h (Fig. 5D). However, the addition of enzyme at 12-h intervals did not lead to a significant increase in the conversion rate. Furthermore, previous studies have suggested that the attachment of enzymes to a substrate surface can limit access to the substrate for other enzymes (Zhang et al., 2015), or that the reaction system can produce unknown intermediates that prevent the reaction from continuing, so that the degradation rate of the crude chitin powder was low.

In order to investigate potential applications for the chitin oligosaccharides, the products were lyophilized and then re-dissolved and serially diluted to different concentrations (50, 75, 100, 125 and 150 mg/mL) to test their antibacterial activity against the Gram-positive bacterial strain *Bacillus subtilis* WB600 and the Gram-negative soil bacterium *Xanthomonas* sp. We found that the chitin oligosaccharide degradation products could obviously inhibit the growth of both *Bacillus subtilis* and *Xanthomonas* sp. at concentrations as low as 50 mg/mL (Fig. 5E). Moreover, the relationship between inhibitory effect and concentration was linear and dose dependent, and the higher the concentration of chitin oligosaccharide, the larger the inhibition zone, indicating greater antibacterial effect. As shown in Fig. 5 F and G, the correlation coefficients reached 0.941 and 0.882, respectively, implying that these chitosan oligosaccharide products could be potentially applied as antibacterial agents.

## Conclusion

In this study, we developed a tool based on the deep learning approaches for discriminating the thermal proteins. The chitinase Chi304 which has maximum activity at 85℃ and showed excellent thermal stability at 80 and 90 °C was screened out. Chi304 had both endo- and exo-activities and the degradation products had good antibacterial activity. The product yields of colloidal chitin degradation reached 97% within 2.5 h, and 20% over 4 days of reaction with crude chitin powder. Thus the novel thermophilic chitinase, Chi304, is of great importance for the industrial biodegradation of chitin.

### Supplementary Information

**Additional file 1: Table S1.** Genome information. **Table S2.** Chitinase_prediction.

**Additional file 2: Figure S1.** Experimental verification of DNS inactivation Chi304. **Figure S2.** Correlation between the frequency of amino acids and thermophilicity. The blue dot indicates that the data is close to the fitted line, the red dot indicates that the data is far away from the fitted line, and the green dot indicates that the data is centered from the fitted line. **Figure S3.** The SDS-PAGE of Chi304. **Figure S4.** The optimum pH and pH stability of Chi304. A: optimum pH; B: pH stability. **Figure S5.** The changes in the physicochemical state of the original crude chitin powder by different pre-treatments. A: ultrasonication (20 min); B: ultrasonic cleaning (20 min); C: microwave irradiation (2 min). **Figure S6.** The changes in the physicochemical state of the original crude chitin powder. A: the original crude chitin powder; B: water bath (80℃, 8 h); C: enzymatic hydrolysis by Chi304 (8 h).

## Data Availability

The authors can confirm that all relevant data are included in the article and/or its Additional files.
