# Supplementary material for: A novel thermophilic chitinase directly mined from the marine metagenome using the deep learning tool Preoptem
Source: Bioresour Bioprocess. 2022 May 16;9(1):54. doi: 10.1186/s40643-022-00543-1 (PMC10991277; doi:10.1186/s40643-022-00543-1)
Supplement: Supplementary file 2 — Additional file 2: Figure S1. Experimental verification of DNS inactivation Chi304. Figure S2. Correlation between the frequency of amino acids and thermophilicity. The blue dot indicates that the data is close to the fitted line, the red dot indicates that the data is far away from the fitted line, and the green dot indicates that the data is centered from the fitted line. Figure S3. The SDS-PAGE of Chi304. Figure S4. The optimum pH and pH stability of Chi304. A: optimum pH; B: pH stability. Figure S5. The changes in the physicochemical state of the original crude chitin powder by different pre-treatments. A: ultrasonication (20 min); B: ultrasonic cleaning (20 min); C: microwave irradiation (2 min). Figure S6. The changes in the physicochemical state of the original crude chitin powder. A: the original crude chitin powder; B: water bath (80℃, 8 h); C: enzymatic hydrolysis by Chi304 (8 h). [file 40643_2022_543_MOESM2_ESM.doc]

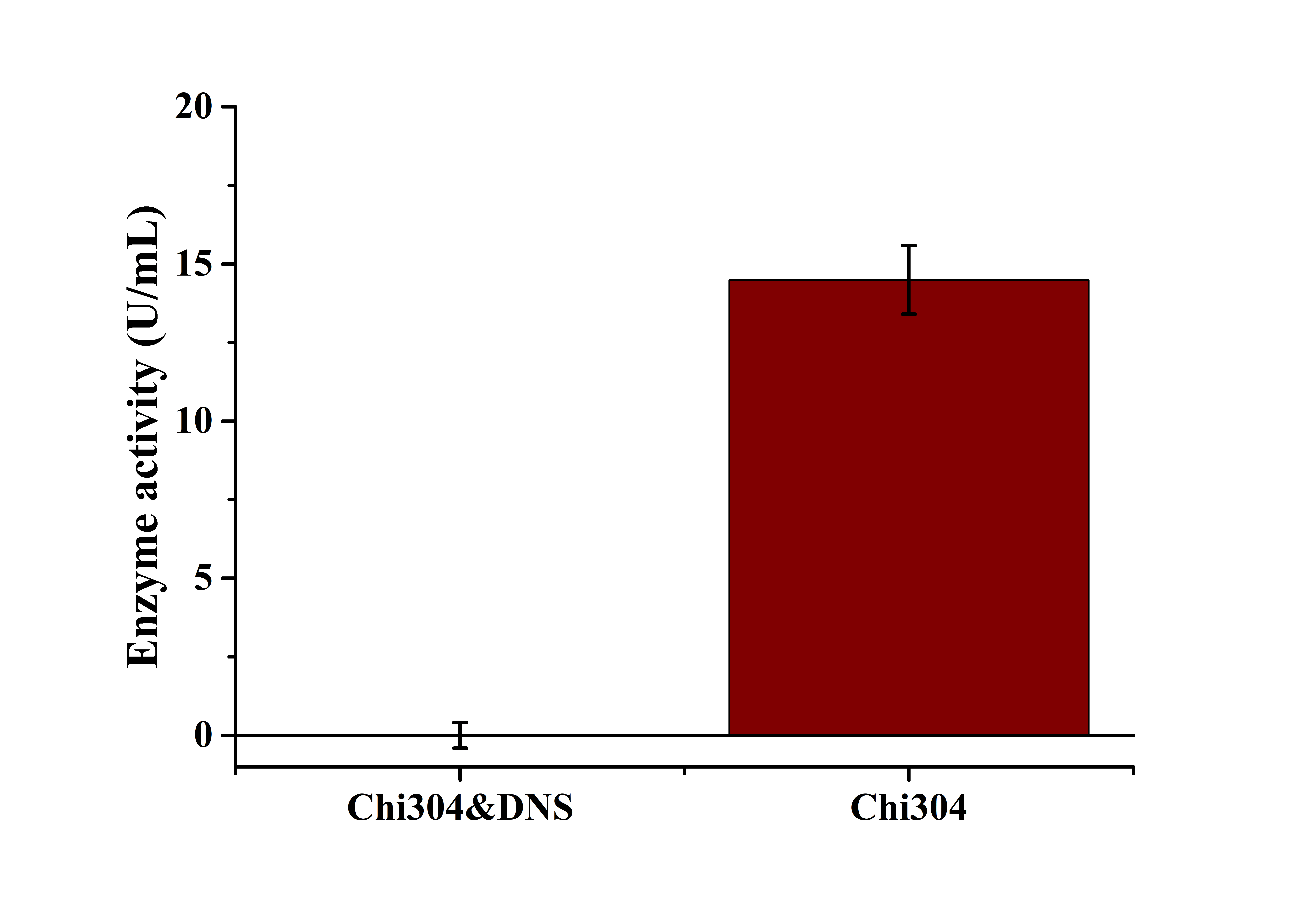


Fig. S1 Experimental verification of DNS inactivation Chi304





Fig. S2 Correlation between the frequency of amino acids and thermophilicity

The blue dot indicates that the data is close to the fitted line, the red dot indicates that the data is far away from the fitted line, and the green dot indicates that the data is centered from the fitted line.


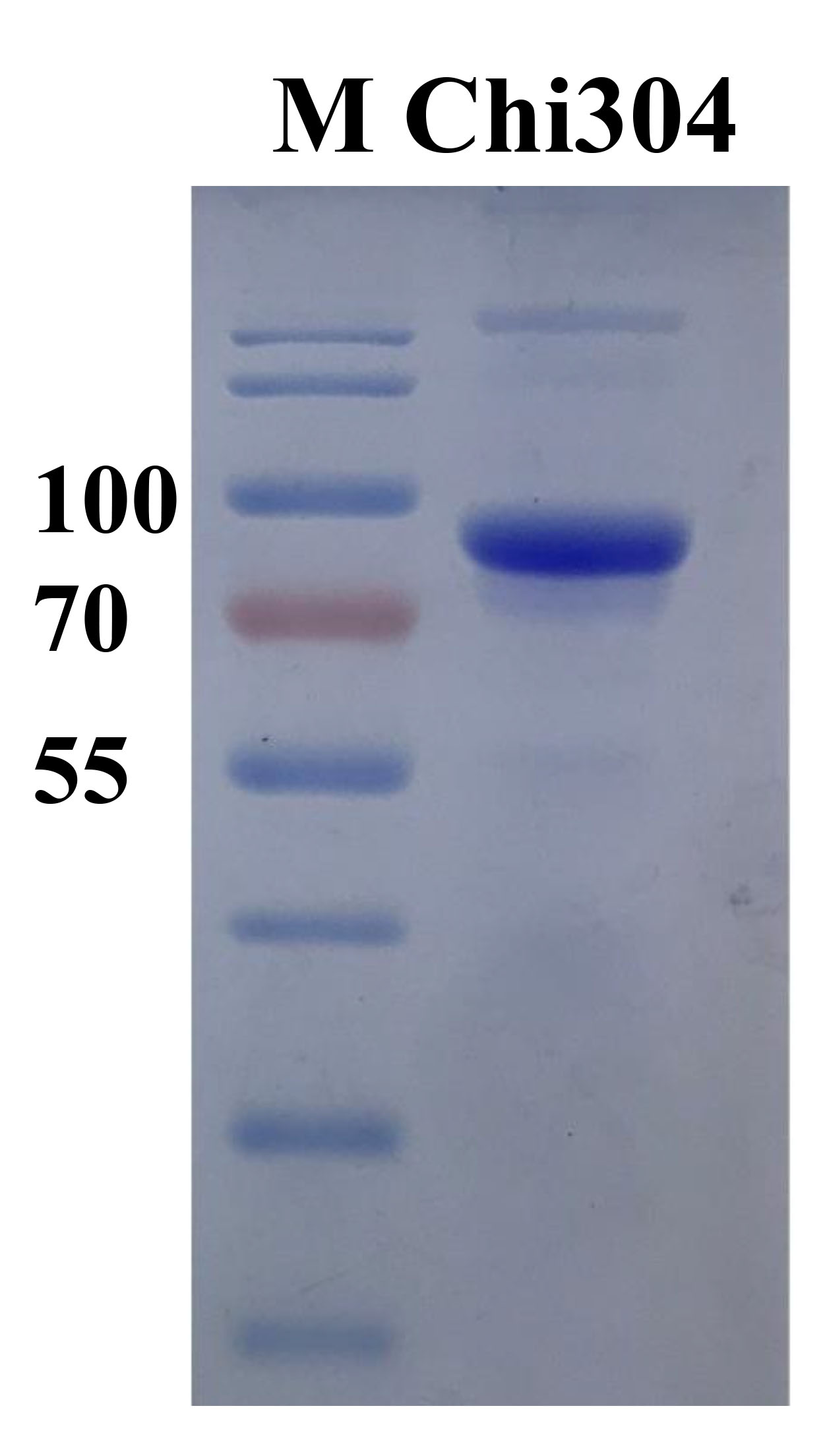


Fig. S3 The SDS-PAGE of Chi304


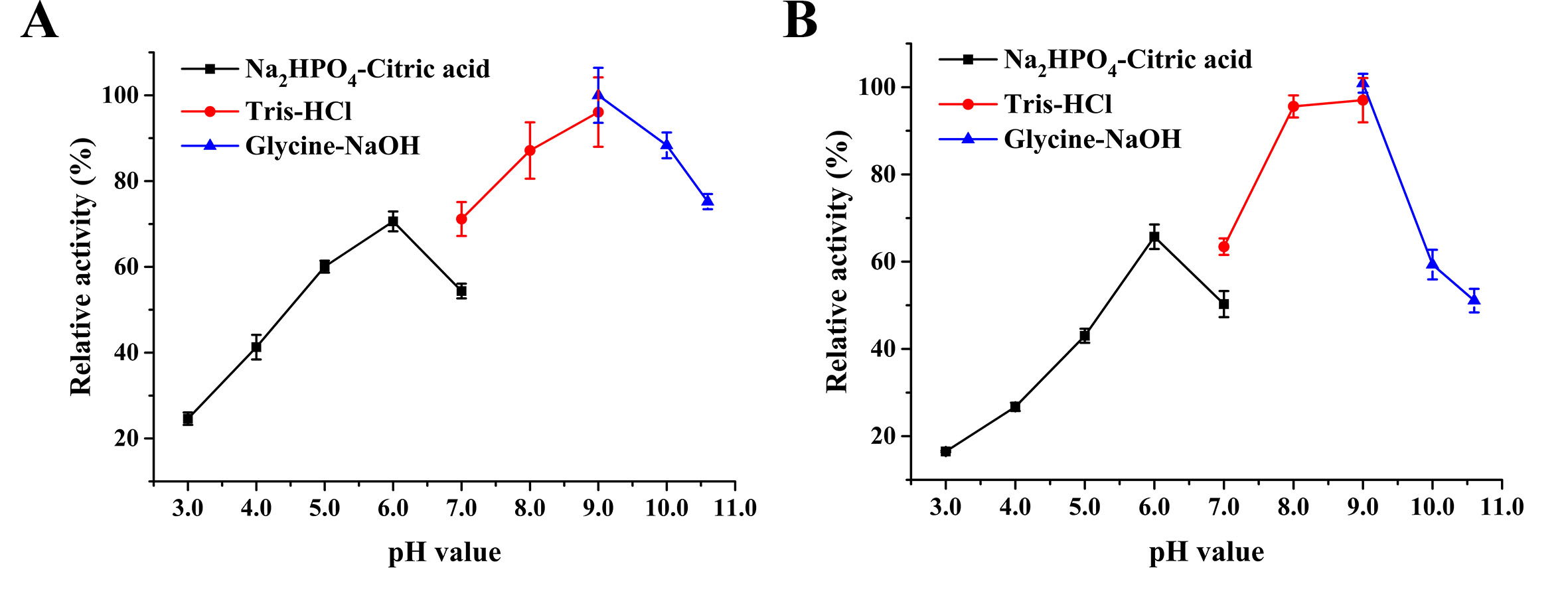


Fig. S4 The optimum pH and pH stability of Chi304

A: optimum pH; B: pH stability.


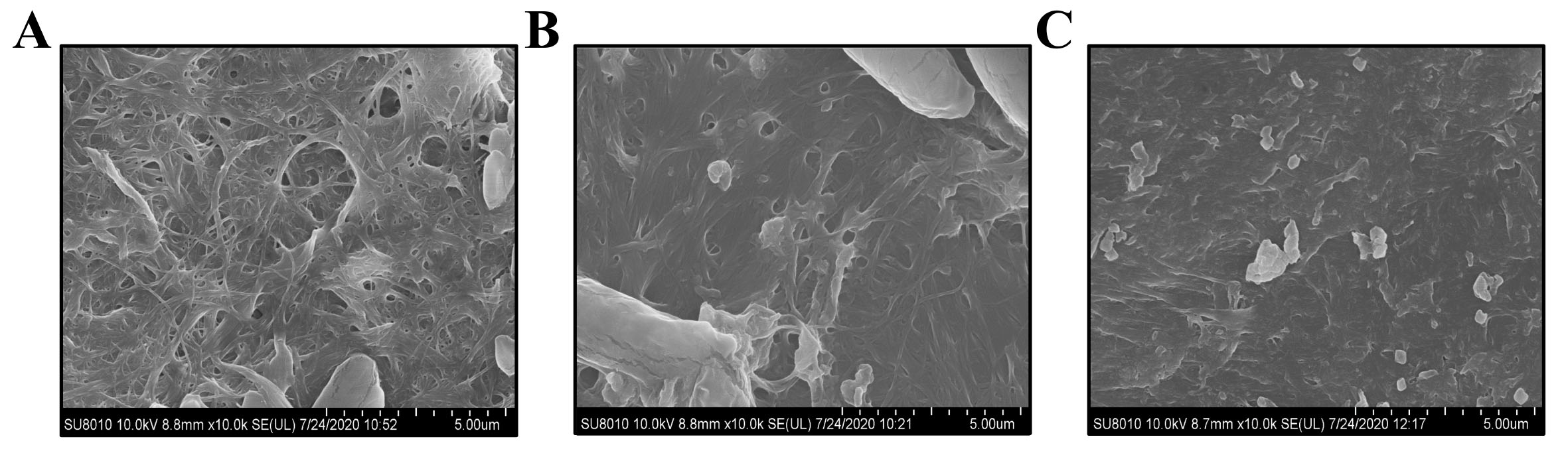


Fig. S5 The changes in the physicochemical state

of the original crude chitin powder by different pre-treatments

A: [ultrasonication](javascript:;) (20 min); B: ultrasonic cleaning (20 min);

C: microwave irradiation (2 min).


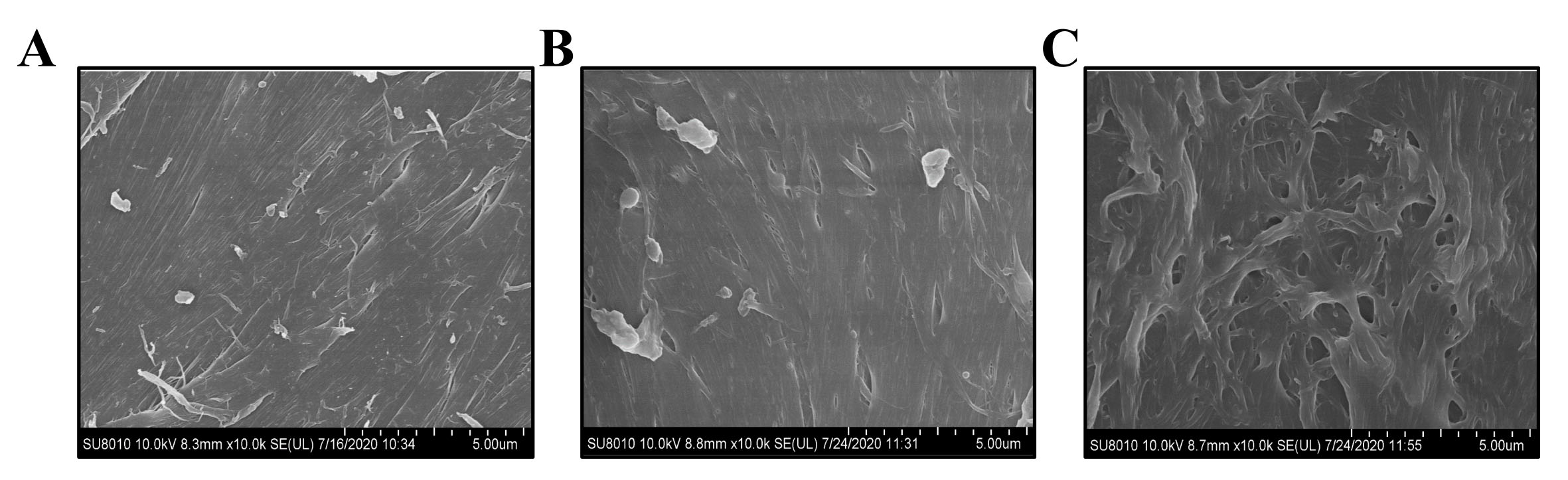


Fig. S6 The changes in the physicochemical state

of the original crude chitin powder

A: the original crude chitin powder; B: water bath (80℃, 8 h);

C: enzymatic hydrolysis by Chi304 (8 h).
